# Interaction Effects of Diabetes and Depression on Hypertension Among US Adults: A Cohort Study

**DOI:** 10.7759/cureus.93792

**Published:** 2025-10-03

**Authors:** Lifeng Zhao, Haisheng Chen

**Affiliations:** 1 Department of Cardiovascular Surgery, Guangzhou First People’s Hospital School of Medicine, South China University of Technology, Guangzhou, CHN

**Keywords:** depression, diabetes, hypertension, interaction, nhanes

## Abstract

Background: Hypertension, depression, and diabetes are major public health concerns that are closely interlinked. This study examined the independent associations of depression and diabetes with hypertension, as well as their combined effects, using data from the National Health and Nutrition Examination Survey (NHANES) between 2005 and 2018.

Methods: Data from NHANES 2005-2018 were analyzed for diabetes, depression, hypertension, and potential confounders. Weighted multivariate logistic regression models were used to assess the associations of depression and diabetes with hypertension, with subgroup analyses by depression severity. Additive interaction effects of depression and diabetes on hypertension were evaluated using the relative excess risk due to interaction (RERI), attributable proportion (AP), and synergy index (SI).

Results: A total of 29,747 participants were included, of whom 10,682 (35.9%) had hypertension. Diabetes was associated with a significantly higher risk of hypertension (odds ratio (OR) = 2.67, 95% confidence interval (CI): 2.40-2.96). Depression was also associated with an increased risk of hypertension (OR = 1.69, 95% CI: 1.48-1.94). Significant additive interaction effects were observed between depression and diabetes on hypertension risk (RERI = 0.75, 95% CI: 0.01-0.48; AP = 0.20, 95% CI: 0.04-0.36; SI = 1.38, 95% CI: 1.04-1.82).

Conclusion: Depression and diabetes demonstrated a synergistic interaction in increasing the risk of hypertension, particularly in patients with moderate depression. These findings highlight the importance of integrated management of mental health and diabetes to prevent or mitigate hypertension.

## Introduction

Hypertension is a clinical disorder characterized by persistently elevated arterial blood pressure. Approximately 50% of US adults have hypertension, which is a major risk factor for cardiovascular disease (CVD), stroke, Alzheimer’s disease, acute coronary syndromes, and kidney disease [1-4]. The primary goal of hypertension management is to prevent or reduce cardiovascular events and related morbidity and mortality [5]. Identifying risk factors and reliable biological indicators of hypertension is therefore essential to mitigate its adverse health outcomes.

Diabetes has also emerged as a major public health challenge, affecting over 38 million people in the United States, with projections suggesting that nearly 61 million adults will have diabetes by 2050 [6,7]. Hypertension is diagnosed in more than half of individuals with diabetes [8]. The coexistence of these two conditions, along with shared risk factors, substantially increases the risk of cardiovascular disease and is associated with poorer clinical outcomes [7].

Depression is the second leading cause of disability worldwide, accounting for 3.0% of disability-adjusted life years (DALYs) [9]. In the United States, the prevalence of major depressive disorder exceeds 21% among women and 10% among men [10]. Depression is a recognized risk factor for hypertension [11]. NHANES data have shown that hypertensive patients without depression had a 15% lower relative mortality risk compared to those with comorbid depression, even after adjusting for significant confounders [12].

These findings suggest that morbidity and mortality risks are higher when hypertension coexists with either diabetes or depression compared to when these conditions occur alone. Moreover, diabetes is linked to increased psychological vulnerability, particularly depression [13]. However, the direct relationship between hypertension and the combined effects of diabetes and depression has not been thoroughly investigated. Therefore, this study examined both the independent and joint effects of diabetes and depression on hypertension using NHANES data from 2005 to 2018.

## Materials and methods

Study population

NHANES is a stratified, multistage, cross-sectional survey of the US population designed to assess the prevalence of chronic and infectious diseases and related risk factors. Since 1999, NHANES has been conducted every two years with a new set of participants in each cycle, using mobile medical examination units and in-home interviews. The survey is authorized by the National Center for Health Statistics Research Ethics Review Board, and all participants provided written informed consent. This study analyzed data from seven NHANES cycles conducted between 2005 and 2018.

**Figure 1:**
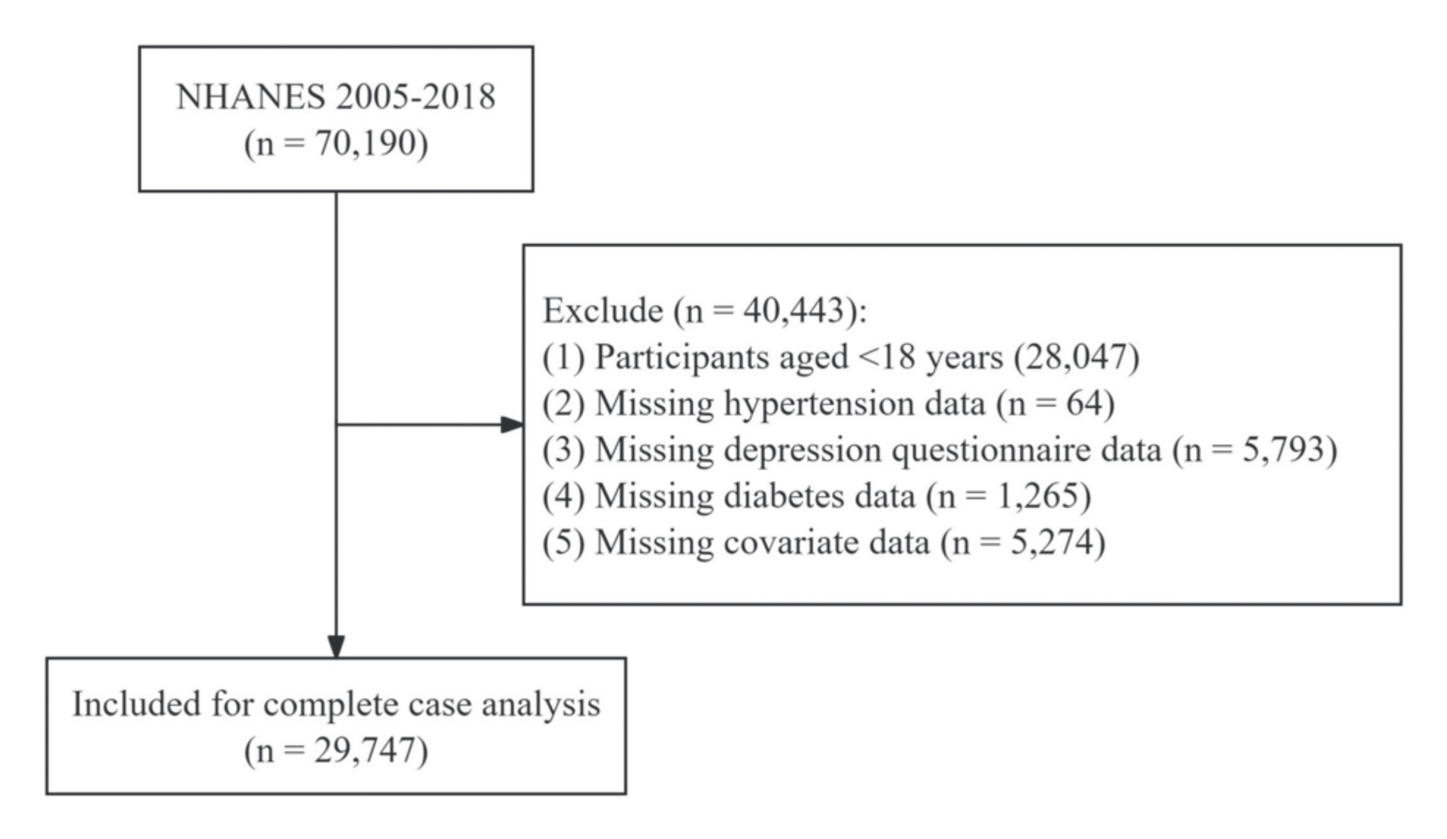
Flowchart of the selection of study participants.

A total of 70,190 participants were initially identified from the NHANES database. Of these, 40,443 were excluded for the following reasons: age <18 years (n = 28,047); missing diabetes data (n = 1,265); missing hypertension data (n = 64); missing depression questionnaire data (n = 5,793); and missing covariate data (n = 5,274). The final analytic sample included 29,747 participants. The flowchart of the selection procedure is shown in Figure 1.

Definition of hypertension

The NHANES team included trained examiners who measured participants’ blood pressure (BP) following the American Heart Association protocol. Hypertension status was also self-reported based on participants’ response (“Yes”) to the question: “Has a doctor ever told you that you have hypertension or high BP?” Although self-reporting is not considered a formal diagnosis, previous epidemiological studies have shown it to be a reliable method for identifying individuals with hypertension. For each participant, the mean value of three BP measurements was calculated. In this study, hypertension was defined as systolic BP ≥ 140 mmHg and/or diastolic BP ≥ 90 mmHg, according to the International Society of Hypertension.

Definition of diabetes and depression

Diabetes was defined according to the 2021 American Diabetes Association (ADA) criteria: (1) HbA1c ≥ 6.5%; (2) blood glucose ≥ 11.1 mmol/L during an oral glucose tolerance test (OGTT); (3) fasting blood glucose ≥ 7.0 mmol/L in laboratory testing; or (4) self-reported history of diabetes.

Depression was assessed using the Patient Health Questionnaire-9 (PHQ-9), a validated tool widely applied in primary care and other clinical settings. The PHQ-9 consists of nine items, with total scores ranging from 0 to 27. A score ≥10 was used as the threshold for clinically significant depression. Based on severity, participants were further classified into three categories: moderate (10-14 points), moderately severe (15-19 points), and severe (≥20 points).

Covariates

Based on previous reports, the following potential confounders for hypertension were included in the analysis: poverty income ratio (PIR), race, sex, education level, age, marital status, body mass index (BMI), alcohol consumption, smoking status, moderate physical activity, sleep duration, stroke, and coronary heart disease. BMI was calculated as weight (kg) divided by height squared (m²) and categorized into three groups: <25.0 kg/m², 25-30 kg/m², and >30 kg/m². Marital status was classified as married, separated, divorced, widowed, never married, or living with a partner. Education level was categorized as below high school, high school, or above high school. Smoking status was classified as never, current, or former. Subjects who had smoked fewer than 100 cigarettes in their lifetime were considered non-smokers; those who had smoked at least 100 cigarettes and were currently smoking were classified as current smokers; and those who had smoked at least 100 cigarettes but had quit were classified as former smokers. Alcohol consumption was dichotomized based on whether participants reported having at least 12 alcoholic drinks per year (yes/no). Sleep duration was assessed by the question: “How much sleep do you usually get at night on weekdays or workdays?” Moderate physical activity was assessed by the question: “Did you do moderate activities for at least 10 minutes that caused only light sweating or a slight to moderate increase in breathing or heart rate?” (yes/no). History of stroke and coronary heart disease was based on participants’ responses to the question: “Have you ever been told by a medical professional that you had ___?”

Statistical analysis

We applied survey weights for each group using primary sampling units, strata, and the two-year sample weights (WTMEC2YR), following NHANES guidelines. Continuous variables were expressed as mean ± standard deviation (SD) or median (interquartile range, IQR), and categorical variables were presented as counts and percentages. Between-group comparisons were performed using analysis of variance (ANOVA) for continuous variables and the chi-squared test for categorical variables. Weighted multivariate logistic regression models were used to evaluate the independent associations of diabetes, depression, and hypertension. To test the robustness of results, three models were constructed: Model 1, unadjusted; Model 2, adjusted for age, sex, race, education level, marital status, and poverty income ratio (PIR) (PIR was log-transformed to approximate normal distribution before inclusion); and Model 3, further adjusted for alcohol consumption, smoking status, BMI, sleep duration, moderate physical activity, stroke, and coronary heart disease. An additive interaction model was used to assess whether diabetes and depression jointly increased the risk of hypertension. Measures of interaction included the relative excess risk due to interaction (RERI), attributable proportion (AP), and synergy index (SI). A joint effect was considered present if the combined risk exceeded the sum of the individual risks of diabetes and depression. No interaction was assumed if the 95% CI for RERI, AP, and SI included 0 or 1. Subgroup analyses were also performed according to depression severity. Statistical analyses were conducted using R software (version 3.3.2) and Free Statistics software (version 1.9.1).

## Results

Description of the study population

This study included 29,747 subjects from the NHANES database (Figure 1). The overall prevalence of hypertension was 35.91% (n = 10,682; Table 1). Compared with normotensive subjects, those with hypertension were more likely to be older, widowed or divorced, less physically active, and to have higher BMI, depression, diabetes, stroke, or coronary heart disease (Table 1).

**Table 1:** Characteristics of the study population. PIR: poverty income ratio, IQR: interquartile range, BMI: body mass index, SD: standard deviation.

Variables	Total (n = 29,747, weighted%)	Groups		p-value
		No hypertension (n = 19,065)	With hypertension (n = 10,682)	
Age (years), n (%)				<0.001
<60	19,859 (74.25)	15,225 (84.35)	4,634 (52.72)	
≥60	9,888 (25.75)	3,840 (15.65)	6,048 (47.28)	
Gender, n (%)				0.347
Male	14,564 (48.59)	9,373 (48.75)	5,191 (48.27)	
Female	15,183 (51.41)	9,692 (51.25)	5,491 (51.73)	
Race, n (%)				<0.001
Mexican American	4,531 (8.02)	3,312 (9.37)	1,219 (5.14)	
Other Hispanic	2,691 (5.10)	1,824 (5.66)	867 (3.91)	
Non-Hispanic White	13,259 (69.44)	8,352 (68.31)	4,907 (71.84)	
Non-Hispanic Black	6,176 (10.40)	3,352 (9.14)	2,824 (13.07)	
Other race	3,090 (7.05)	2,225 (7.52)	865 (6.04)	
Educational level, n (%)				<0.001
Below high school	6,889 (14.84)	4,140 (13.83)	2,749 (16.98)	
High school	6,863 (23.18)	4,208 (22.14)	2,655 (25.42)	
Above high school	15,995 (61.98)	10,717 (64.03)	5,278 (57.60)	
Marital status, n (%)				< 0.001
Married	15,496 (56.06)	9,795 (54.82)	5,701 (58.70)	
Widowed	2,268 (5.52)	795 (2.91)	1,473 (11.07)	
Divorced	3,325 (10.51)	1,818 (9.40)	1,507 (12.88)	
Separated	973 (2.32)	586 (2.20)	387 (2.57)	
Never married	5,243 (17.43)	4,200 (21.39)	1,043 (8.97)	
Living with partner	2,442 (8.16)	1,871 (9.27)	571 (5.80)	
PIR, median (IQR)	2.15 (1.13, 4.12)	2.20 (1.13, 4.22)	2.06 (1.12, 3.91)	<0.001
BMI (kg/m^2^), n (%)				<0.001
<25	8,420 (29.32)	6,587 (35.47)	1,833 (16.20)	
25–30	9,823 (32.92)	6,425 (33.48)	3,398 (31.71)	
≥30	11,504 (37.76)	6,053 (31.04)	5,451 (52.08)	
Alcohol consumption, n (%)				<0.001
No	7,600 (20.24)	4,573 (18.82)	3,027 (23.27)	
Yes	22,147 (79.76)	14,492 (81.18)	7,655 (76.73)	
Smoking status, n (%)				<0.001
Now	6,146 (20.18)	4,151 (20.93)	1,995 (18.57)	
Never	16,264 (54.74)	10,986 (57.38)	5,278 (49.12)	
Former	7,337 (25.08)	3,928 (21.69)	3,409 (32.31)	
Moderate activity, n (%)				< 0.001
No	17,734 (54.73)	11,013 (53.46)	6,721 (57.44)	
Yes	12,013 (45.27)	8,052 (46.54)	3,961 (42.56)	
Sleep duration (h), mean ± SD	7.05 ± 1.51	7.07 ± 1.45	7.02 ± 1.61	0.001
Depression, n (%)				<0.001
No	27,164 (92.38)	17,703 (93.72)	9,461 (89.51)	
Yes	2,583 (7.62)	1,362 (6.28)	1,221 (10.49)	
Diabetes, n (%)				<0.001
No	24,300 (86.37)	17,163 (92.91)	7,137 (72.44)	
Yes	5,447 (13.63)	1,902 (7.09)	3,545 (27.56)	
Stroke, n (%)				<0.001
No	28,660 (97.29)	18,813 (98.99)	9,847 (93.66)	
Yes	1,087 (2.71)	252 (1.01)	835 (6.34)	
Coronary heart disease, n (%)				<0.001
No	28,537 (96.60)	18,787 (98.71)	9,750 (92.11)	
Yes	1,210 (3.40)	278 (1.29)	932 (7.89)	

Correlation of hypertension with diabetes

**Figure 2:**
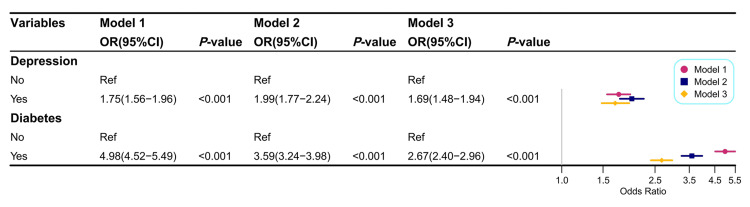
Weighted multivariate logistic regression models for analyzing the relationship of depression and diabetes with hypertension. Note: Model 1: not adjusted. Model 2: adjusted by age, gender, race, educational level, marital status, and PIR. Model 3: adjusted by age, gender, race, educational level, marital status, PIR, BMI, alcohol consumption, smoking status, moderate activity, sleep duration, stroke, and coronary heart disease.

Patients with diabetes had a significantly higher risk of developing hypertension (Model 1, unadjusted OR: 4.98; 95% CI: 4.52-5.49; Figure 2). After adjusting for demographic and socioeconomic factors (Model 2), the association remained significant (adjusted OR: 3.59; 95% CI: 3.24-3.98). Even after further adjustment for BMI, alcohol intake, smoking status, physical activity, sleep duration, stroke, and coronary heart disease (Model 3), diabetics continued to show a markedly elevated risk of hypertension (adjusted OR: 2.67; 95% CI: 2.40-2.96; Figure 2).

Association of depression with hypertension

As shown in Figure 2, depression was significantly associated with an increased risk of hypertension (Model 3, adjusted OR: 1.69; 95% CI: 1.48-1.94).

Interaction effects of diabetes and depression on hypertension

**Figure 3:**
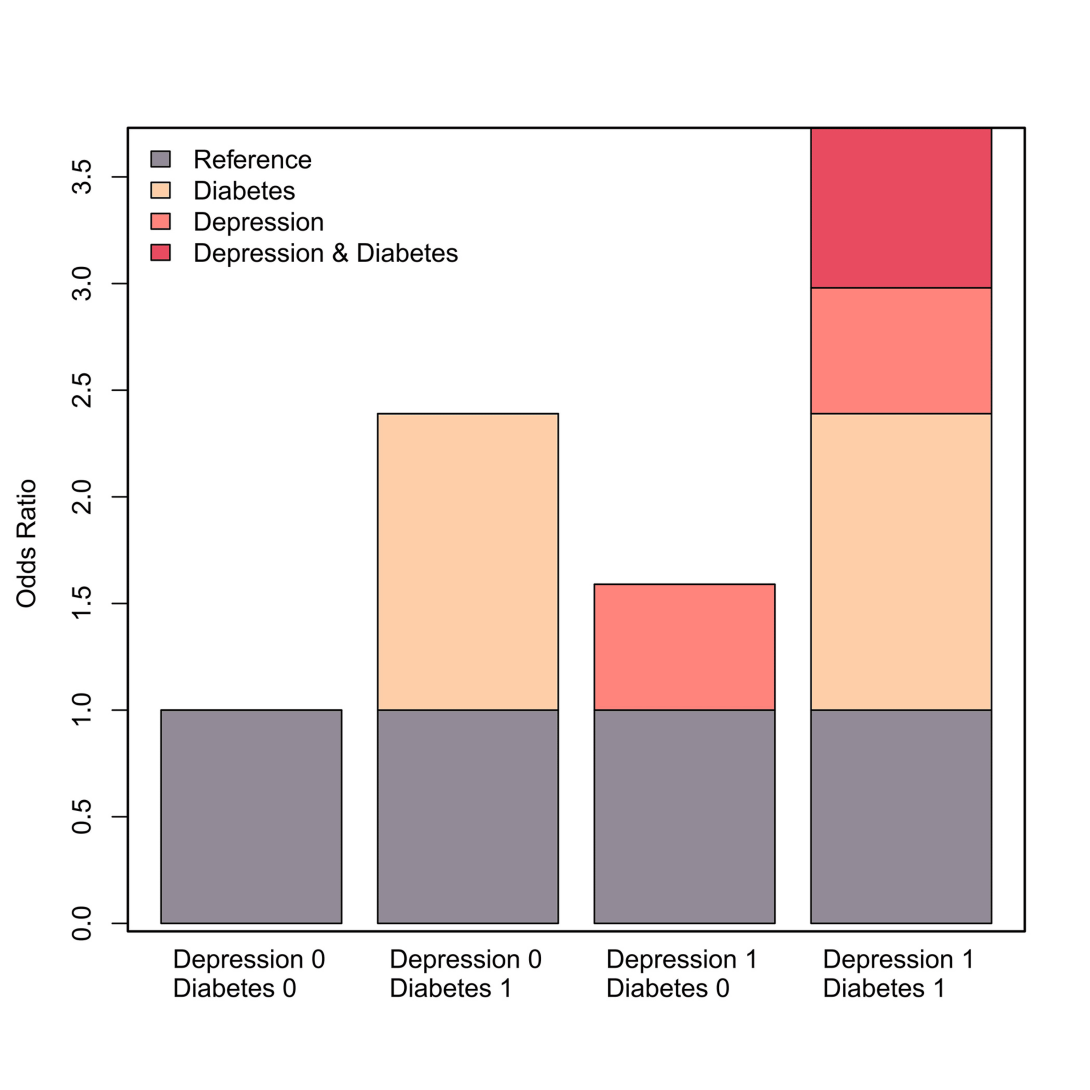
Visual analysis of the additive interactions between risk factors for hypertension in patients with diabetes and depression.

Diabetes and depression demonstrated a significant synergistic effect on the risk of hypertension (Model 3: RERI = 0.75, 95% CI: 0.01-0.48; AP = 0.20, 95% CI: 0.04-0.36; SI = 1.38, 95% CI: 1.04-1.82) (Table 2). The AP value indicated that the combined effect of diabetes and depression accounted for 16.6% of hypertension cases in this study. Overall, the interaction effect of diabetes and depression on hypertension risk was greater than the sum of their individual effects (Figure 3).

**Table 2:** Association of diabetes with depression and hypertension. RERI: relative excess risk due to interaction, AP: attributable proportion of interaction, SI: synergy index. Model 1: not adjusted. Model 2: adjusted by age, gender, race, educational level, marital status, and PIR. Model 3: adjusted by age, gender, race, educational level, marital status, PIR, BMI, alcohol consumption, smoking status, moderate activity, sleep duration, stroke, and coronary heart disease.

Depression	Diabetes	Hypertension/total (n)	Model 1			Model 2			Model 3		
			OR	95% CI	p-value	OR	95% CI	p-value	OR	95% CI	p-value
0	0	6,406/22,386	Ref	-	-	Ref	-	-	Ref	-	-
0	1	3,055/4,778	4.42	4.14-4.72	<0.001	3.03	2.82-3.26	<0.001	2.39	2.22-2.57	<0.001
1	0	731/1,914	1.54	1.4-1.7	<0.001	1.8	1.62-2	<0.001	1.59	1.42-1.77	<0.001
1	1	490/669	6.83	5.74-8.12	<0.001	5.66	4.7-6.81	<0.001	3.72	3.08-4.51	<0.001
RERI (95% CI)			1.86 (0.66-3.07)	-	-	1.82 (0.76-2.88)	-	-	0.75 (0.01-1.48)	-	-
AP (95% CI)			0.27 (0.14-0.4)	-	-	0.32 (0.19-0.45)	-	-	0.2 (0.04-0.36)	-	-
SI (95% CI)			1.47 (1.19-1.82)	-	-	1.64 (1.29-2.09)	-	-	1.38 (1.04-1.82)	-	-

Interaction effect of diabetes and depression severity on hypertension

After adjusting for multiple confounding factors in Model 3, the combination of diabetes and moderate depression was significantly associated with an increased risk of hypertension (RERI = 1.45, 95% CI: 0.38-2.52; AP = 0.34, 95% CI: 0.17-0.51; SI = 1.81, 95% CI: 1.27-2.56). These findings indicate a synergistic relationship between moderate depression and diabetes in relation to hypertension risk (Table 3).

**Table 3:** Association of diabetes with depression severity and hypertension. RERI: relative excess risk due to interaction, AP: attributable proportion of interaction, SI: synergy index.

Depression	Diabetes	Moderate depression			Moderately severe depression			Severe depression		
		OR	95% CI	p-value	OR	95% CI	p-value	OR	95% CI	p-value
0	0	Ref	-	-	Ref	-	-	Ref	-	-
0	1	2.38	2.21-2.57	<0.001	2.38	2.21-2.56	<0.001	2.38	2.21-2.56	<0.001
1	0	1.41	1.23-1.62	<0.001	1.94	1.59-2.36	<0.001	2.04	1.49-2.8	<0.001
1	1	4.25	3.31-5.45	<0.001	3.1	2.19-4.39	<0.001	3.11	1.84-5.26	<0.001
RERI (95% CI)		1.45 (0.38-2.52)	-	-	-0.21 (-1.36 to 0.93)	-	-	-0.31 (-2.07 to 1.45)	-	-
AP (95% CI)		0.34 (0.17-0.51)	-	-	-0.07 (-0.46 to 0.32)	-	-	-0.1 (-0.71 to 0.51)	-	-
S (95% CI)		1.81 (1.27-2.56)	-	-	0.91 (0.53-1.56)	-	-	0.87 (0.38-1.98)	-	-

## Discussion

This study analyzed data from 29,747 adults in the NHANES database (2005-2018) to examine the associations of diabetes and depression with hypertension and their potential interaction. The findings showed that diabetes and depression, particularly moderate depression, had a significant synergistic effect on the risk of hypertension, exceeding the impact of each condition alone.

After adjusting for multiple variables such as sex, race, age, education level, marital status, PIR, BMI, alcohol intake, smoking history, moderate physical activity, sleep duration, stroke, and coronary heart disease, we observed a significant positive association between diabetes and hypertension in the study cohort. This finding is consistent with previous studies reporting a strong correlation between the two conditions. Diabetes influences blood pressure regulation through several mechanisms involving cardiac output and systemic vascular resistance (SVR). In diabetes, increased sodium and water retention expand blood volume, which in turn elevates stroke volume and cardiac output. Endothelial dysfunction caused by oxidative stress and inflammation further impairs vascular relaxation. At the same time, sympathetic nervous system activation in diabetic patients raises SVR by constricting blood vessels. Progressive arterial stiffening and narrowing further increase vascular resistance, contributing to sustained hypertension. The coexistence of diabetes and hypertension is associated with worse outcomes, as hypertension amplifies the risk of cardiovascular and renal complications [14]. Several studies have demonstrated that this combination increases the likelihood of stroke, heart failure, and kidney failure [15,16]. Effective blood pressure control is therefore essential to reduce the risk of macrovascular events (e.g., stroke), as well as microvascular complications such as microalbuminuria and retinopathy [17].

Diabetes is a complex chronic disorder often accompanied by mental health problems, including psychological disorders [18]. Song et al. used the support vector machine (SVM) learning algorithm to analyze blood tests and vital signs, demonstrating a significant correlation between depression and increased blood pressure (BP) [9]. Similarly, two cohort studies identified depression as a significant risk factor for developing hypertension [19,20]. These findings align with our study, in which the fully adjusted model also showed a significant positive association between depression and hypertension. Previous research has highlighted strong correlations between hypertension and comorbid conditions such as diabetes and depression [21]. Zhang et al. further reported that depression could act as a trigger for hypertension, which in turn may worsen depressive symptoms [22]. Offidani et al. described a vicious cycle in hypertensive patients, where elevated BP, anxiety, depression, and other negative emotions reinforce one another through repeated illness episodes [23]. Therefore, effective treatment and management strategies are essential, not only to stabilize BP but also to improve mood and break this cycle in patients with hypertension.

Depression contributes to the progression of both diabetes and hypertension by promoting inflammation, encouraging unhealthy lifestyle behaviors, and increasing non-compliance with medical therapies [24]. It also worsens the quality of life in individuals with diabetes by impairing functional capacity, cognitive performance, and psychological well-being [25]. Depressive symptoms are further linked to higher blood glucose levels in patients with diabetes [26]. For instance, Bot et al., in a study conducted across three tertiary diabetes clinics in the Netherlands, found that several depressive symptoms were significantly associated with elevated HbA1c levels [27]. Diabetes itself has been associated with an increased risk of hypertension. Gray et al., in a long-term study of community-dwelling older adults in Alabama, reported a clear link between diabetes and the co-occurrence of depression and hypertension [28]. Consistent with these findings, our data demonstrated that diabetes and depression synergistically increase the risk of hypertension. Importantly, psychological stability and higher levels of happiness are beneficial for glycemic control, while depressive states and other adverse psychosocial factors negatively impact blood glucose levels [29]. Negative mood represents a significant risk factor for both the physical and emotional health of patients with diabetes. Our subgroup analysis further revealed that the synergistic effect of moderate depression and diabetes was strongly associated with increased blood pressure. Although the associations with moderately severe or severe depression did not reach statistical significance, the combined interaction effect of these conditions was still greater than their individual effects. Therefore, healthcare professionals should proactively address psychological disorders, especially depressive symptoms, when managing patients with diabetes, with particular emphasis on those receiving ongoing treatment for hypertension.

This study has several notable strengths. First, we analyzed seven cycles of nationally representative data from US participants in the NHANES database, ensuring a large sample size and broad generalizability. Second, although prior studies have shown that diabetes and depression are independent risk factors for hypertension, few have examined their potential interaction. Our study addressed this gap by demonstrating that the synergistic effect of diabetes and depression was significantly associated with an increased risk of hypertension. Third, we employed logistic regression models with extensive adjustments for potential confounders, including lifestyle, socioeconomic, health, and demographic factors, which strengthened the validity of our findings.

However, several limitations should be acknowledged. The cross-sectional design precludes causal inference, and prospective studies are needed to confirm these associations. Residual confounding may remain despite adjustments, particularly due to measurement errors. In addition, NHANES relies on self-reported data from interviews and questionnaires, which may be subject to recall bias and misclassification. Nonetheless, prior studies have consistently reported strong links between diabetes, depression, and hypertension, supporting the robustness of our findings. Finally, we were unable to differentiate between type 1 and type 2 diabetes, as NHANES does not collect this information. Since type 2 diabetes accounts for approximately 8.5% of cases in the United States compared to only 0.5% for type 1 diabetes, our results are likely more applicable to type 2 diabetes [30].

## Conclusions

Our findings demonstrated that depression and diabetes exerted a synergistic effect on hypertension. Subgroup analysis further revealed that moderate depression and diabetes showed a particularly strong interaction, substantially increasing the risk of hypertension. These results suggest that both blood glucose control and psychological well-being are critical in the management of patients with hypertension. In addition, our epidemiological data confirmed that diabetes and depression were each independently associated with hypertension, alongside their combined synergistic effect.
